# NAD^+^ dyshomeostasis in *RYR1*-related myopathies

**DOI:** 10.1186/s13395-025-00390-6

**Published:** 2025-08-22

**Authors:** Tokunbor A. Lawal, Willa Riekhof, Linda Groom, Pooja Varma, Irene C. Chrismer, Angela Kokkinis, Christopher Grunseich, Jessica W. Witherspoon, Muslima S. Razaqyar, Ninet Sinaii, Katherine G. Meilleur, Lichen Xiang, Jana Buzkova, Liliya Euro, Payam Mohassel, Robert T. Dirksen, Joshua J. Todd

**Affiliations:** 1 Skeletal Myopathies Unit, Translational Biobehavioral and Health Disparities Branch, NIH Clinical Center, National Institutes of Health, Bethesda, MD USA; 2 National Institute of Nursing Research, National Institutes of Health, Bethesda, MD USA; 3 Department of Pharmacology and Physiology, University of Rochester Medical Center, Rochester, NY USA; 4 Inherited Neuromuscular Diseases Unit, National Institute of Neurological Disorders and Stroke, National Institutes of Health, Bethesda, MD USA; 5 Biostatistics and Clinical Epidemiology Service, National Institutes of Health Clinical Center, Bethesda, MD USA; 6 NADMed Ltd, Biomedicum 1, Haartmaninkatu 8, Helsinki, Finland; 7 Johns Hopkins University School of Medicine, Baltimore, MD USA; 8 Neuromuscular and Neurogenetic Disorders of Childhood Section, National Institute of Neurological Disorders and Stroke, National Institutes of Health, Bethesda, MD USA; 9 Clinical Trials Unit, National Institute of Neurological Disorders and Stroke, Bethesda, MD USA

**Keywords:** Congenital myopathy, Oxidative stress, *RYR1*, Glutathione, NAD^+^, NADP

## Abstract

**Background:**

Pathogenic variants in *RYR1* cause a spectrum of rare congenital myopathies associated with intracellular calcium dysregulation. Glutathione redox imbalance has been reported in several *Ryr1* disease model systems and clinical studies. NAD^+^ and NADP are essential cofactors in cellular metabolism and redox homeostasis. NAD^+^ deficiency has been associated with skeletal muscle bioenergetic deficits in mitochondrial myopathy and sarcopenia.

**Methods:**

Using a new colorimetric assay and large control dataset (*n* = 299), we assessed redox balance (glutathione, NAD^+^, and NADP) in whole blood from 28 *RYR1*-RM affected individuals (NCT02362425). Analyses were expanded to human skeletal muscle (*n* = 4), primary myotube cultures (*n* = 5), and whole blood and skeletal muscle specimens from *Ryr1* Y524S mice. The in vitro effects of nicotinamide riboside (NR) on cellular NAD^+^ content and mitochondrial respirometry were also tested.

**Results:**

At baseline, a majority of affected individuals exhibited systemic NAD^+^ deficiency (19/28 [68%] < 21 µM) and increased NADPH concentrations (22/26 [85%] > 1.6 µM). When compared to controls, decreased NAD^+^/NADH and NADP/NADPH ratios were observed in 9/28 and 23/26 individuals, respectively. In patient-derived myotube cultures (*n* = 5), NR appeared to increase cellular NAD^+^ concentrations in a dose and time-dependent manner at 72-h only and favorably modified maximal respiration and ATP production. Average whole blood GSH/GSSG ratio was comparable between groups, and redox imbalance was not observed in *Ryr1* Y524S specimens.

**Conclusions:**

NAD^+^ and NADP dyshomeostasis was identified in a subset of *RYR1*-RM affected individuals. Further experiments are warranted to confirm if NAD^+^ repletion could be an attractive therapeutic approach given the favorable outcomes reported in other neuromuscular disorders.

**Graphical Abstract:**

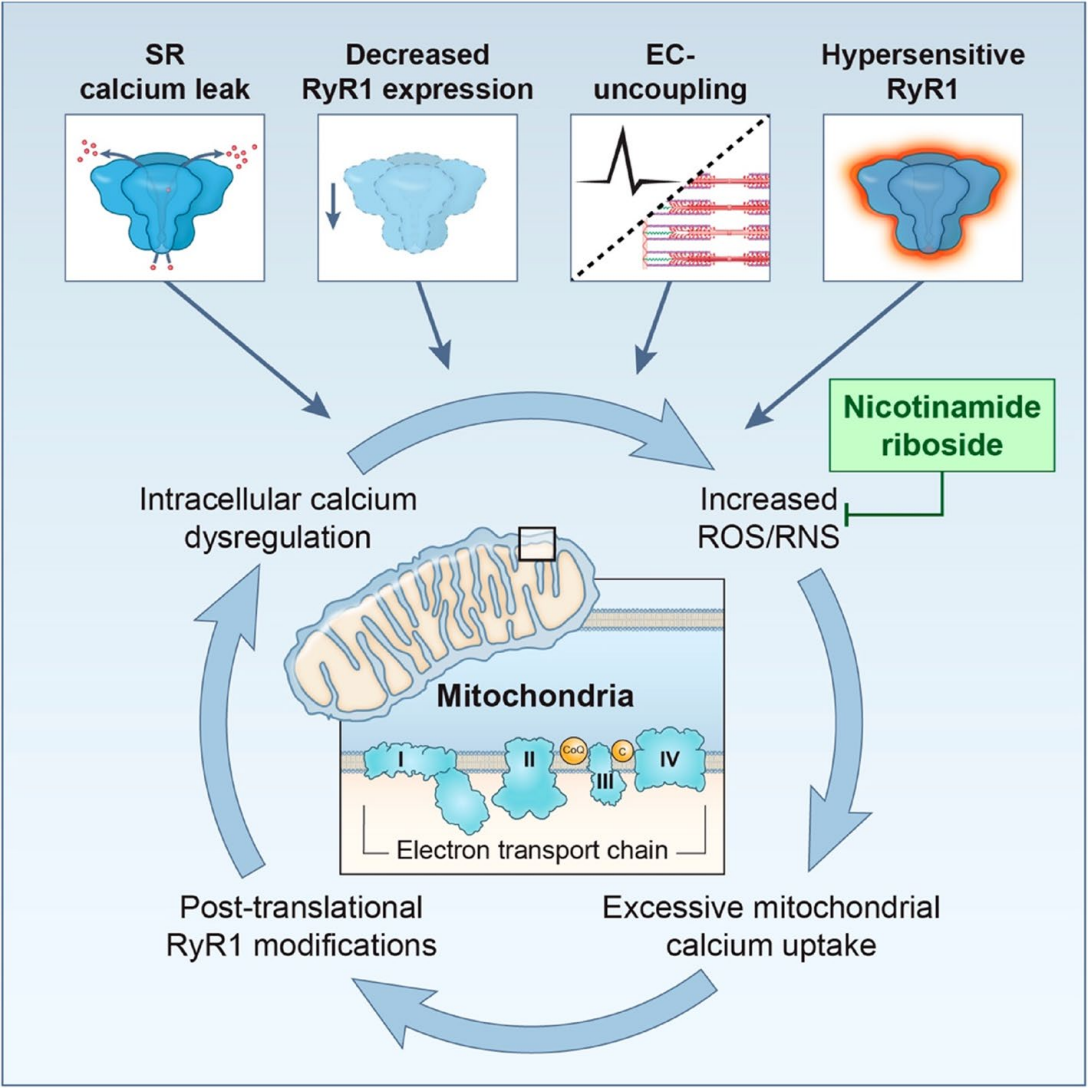

**Supplementary Information:**

The online version contains supplementary material available at 10.1186/s13395-025-00390-6.

## Introduction

The *RYR1* gene (19q13.2) comprises 106 exons that encode for the skeletal muscle ryanodine receptor (RyR1). Pathogenic *RYR1* variants result in a heterogenous spectrum of *RYR1-*related disorders (*RYR1*-RD). These include *RYR1*-related myopathies (*RYR1*-RM) that typically have a static or slowly progressive course and thus result in substantial disease burden over an affected individual’s lifetime. Dominant and recessive cases of *RYR1*-RM have been reported with manifestations including fatigue, proximal muscle weakness, contractures, scoliosis, ophthalmoplegia, and respiratory insufficiency.

Localized to the sarcoplasmic reticulum (SR) membrane, RyR1 is a 2.2 mega-Dalton homotetrameric ion channel which gates and releases SR calcium stores in response to action potentials. RyR1 is critical to excitation–contraction coupling, a process that underpins skeletal muscle contraction [1]. Intracellular calcium dysregulation, RyR1 channel hypersensitivity, and diminished RyR1 protein expression have all been associated with pathogenic *RYR1* variants [2]. In model systems, the resulting oxidative stress has been reported to post-translationally modify RyR1, further perturbing intracellular calcium homeostasis [3]. The RyR1 channel structure is especially susceptible to oxidative stress owing to the presence of highly reactive cysteine residues (100 per monomer), several of which are reported to play a role in RyR1 gating [4, 5]. Oxidation and/or nitrosylation of these cysteine residues has been shown to further perpetuate RyR1 dysfunction [6, 7]. Redox imbalance may be a point of convergence, downstream from different dysfunctional states of the RyR1 channel in *RYR1*-RM (i.e., leaky channel but also decreased RyR1 protein expression), and thus *RYR1*-RM may be broadly amenable to antioxidants as an adjunct therapy [8, 9].

Our prior phase two clinical trial testing the glutathione precursor *N*-acetylcysteine (NAC, NCT02362425), demonstrated that NAC treatment did not favorably modify oxidative stress or endurance in ambulatory individuals with *RYR1*-RM [10]. However, this study had several limitations. Glutathione redox imbalance (decreased GSH:GSSG ratio) was measured using mass spectrometry in *RYR1*-RM trial participants at baseline only, and interpretation was limited by a small otherwise healthy control group (*n* = 24) [10]. Here, we aimed to utilize a newly available redox profiling method based on enzymatic colorimetric assays and an otherwise healthy control dataset (*n* = 299) to re-assess GSH/GSSG ratio in banked whole blood specimens from all NCT02362425 study intervals (Month 0, Month 6, and Month 12). Additionally, we explored the concentrations of reduced and oxidized forms of nicotinamide adenine dinucleotide (NAD^+^, NADH, NADP, and NADPH), which are important cofactors involved in cellular metabolism and cellular redox homeostasis [11], since these were available as part of the new testing panel.

Average whole blood GSH/GSSG ratio was comparable between *RYR1*-RM affected individuals and otherwise healthy controls, deviating from our prior report. In contrast, a majority 19/28 (68%) of *RYR1*-RM affected individuals exhibited systemic NAD^+^ deficiency (< 21 µM) and 2/28 (7%) had increased NADH concentrations (> 1.6 µM). Of the 19 individuals with systemic NAD^+^ deficiency, 9 (47%) also had a diminished NAD^+^/NADH ratio. Based on these preliminary observations and published data supporting that NAD^+^ repletion is efficacious in the *mdx* model of Duchenne Muscular Dystrophy and adult-onset mitochondrial myopathy patients [12, 13], we investigated the in vitro effects of nicotinamide riboside (NR, a vitamin B_3_ derivative) treatment on cellular NAD^+^ content and parameters of mitochondrial respiration. In a small number of *RYR1*-RM primary myotube cultures (*n* = 5), NR appeared to increase cellular NAD^+^ concentrations in a dose and time-dependent manner and favorably modify maximal respiration and ATP production. Additionally, most *RYR1*-RM affected individuals also had an increased systemic NADPH concentration (85%) and decreased NADP/NADPH ratio (88%) when compared to general population norms. These preliminary findings warrant further investigation.

## Subjects and Methods

All individuals provided written informed consent or assent (if applicable) prior to initiating research procedures, all of which were approved by the NIH Intramural IRB (NCT02362425 and NCT04141670). The analysis presented here was conducted using deidentified and unlinked data and biospecimens obtained from individuals who consented to future research use. Primary NAC trial results have been published elsewhere, and full eligibility criteria are available at clinicaltrials.gov (NCT02362425) [10]. Briefly, eligible individuals had a genetic diagnosis of *RYR1*-RM, were ambulatory (able to walk 10 m with or without assistance), and were at least seven years of age. The trial design comprised a six-month natural history lead-in phase (Month 0 to Month 6 interval) after which participants were block randomized (1:1) to receive oral NAC or placebo for six months (through Month 12 interval). NAC (30 mg/kg/day not to exceed 2700 mg daily) and equivalent placebo were provided in a commercially available effervescent tablet formulation. Average compliance to intervention was 96%. Research assessments and biospecimens were obtained at each visit to the NIH Clinical Center, Bethesda, MD, USA.

### Human biospecimen collection and preparation

Human whole blood specimens pre- and post-treatment with NAC or placebo (*n* = 66), were obtained under protocol NCT02362425 by venipuncture, snap frozen on dry ice, and stored at −80 °C until analysis. Skeletal muscle tissue specimens from *RYR1*-RM affected individuals (*n* = 5) were obtained by tibialis anterior needle biopsies at baseline study visits under each protocol. Post-mortem skeletal muscle tissue specimens from otherwise healthy individuals (healthy controls, *n* = 4) were obtained through the National Disease Research Interchange (NDRI). Following collection, fresh muscle tissue was washed in phosphate buffered saline and placed in transport medium (DMEM, penicillin–streptomycin, gentamycin, and amphotericin B). Under sterile conditions, muscle specimens were transferred to 5 mL conditioning medium (Medium 199, fetal bovine serum, penicillin–streptomycin, and amphotericin B) and incubated overnight at 37 ˚C and 5% CO_2_. Following overnight incubation, muscle tissue specimens were transferred to sterile microtubes each containing 1 mL freezing medium (DMEM, DMSO, fetal bovine serum, penicillin–streptomycin, insulin, L-glutamine, and human fibroblast growth factor) and placed in a cryo-freezing container overnight at −80 °C. Finally, cryotubes were transferred to liquid nitrogen for long-term storage.

### Measurement of normal levels of redox analytes

The normal levels for NADs, NADPs and glutathione metabolites were established using blood of 299 otherwise healthy blood donors aged 18–70 years [14]. Frozen blood samples were received from RedCross Blood Service (www.bloodservice.fi) in Finland and target metabolites were measured using proprietary methodology [14]. Donor blood was collected by the Red Cross blood donor service according to ethical permits of the biobank. Donors were informed about the study and gave a written consent prior to blood donation for the study. Aliquots of 200µL of donated blood were frozen and delivered on dry ice for redox measurements. The normal values of NAD +, NADH, NADP, NADPH, GSH and GSSG for frozen blood samples were calculated as mean ± one standard deviation and were determined to be as follows: NAD +, 28.03 ± 6.17 µM; NADH, 1.21 ± 0.39 µM; NADP, 13.20 ± 2.4 µM; NADPH 1.6 ± 0.5 μM; GSH, 760 ± 140 µM; GSSG, 40 ± 10 µM.

### Murine skeletal muscle and whole blood collection

Murine skeletal muscle tissue samples were obtained from a well-characterized strain of mice harboring the pathogenic *Ryr1* variant Y524S associated with malignant hyperthermia susceptibility [15]. Age-matched C57BL/6 wild-type mice were included as controls. Y524S mice were sacrificed at 12 months of age, a timepoint at which this strain has been reported to exhibit mitochondrial damage, core formation, and myofibrillar disruption [16]. Soleus muscle was selected for redox analyses owing to the predominant type II oxidative fiber composition and thus expected higher mitochondrial content. Following sacrifice, soleus skeletal muscle was excised, rinsed in sterile dH_2_O, snap frozen in liquid nitrogen, and stored at −80 Celsius until analysis. Whole blood was obtained from age-matched *Ryr1* Y524S and C57BL/6 wild-type mice upon sacrifice by cardiac puncture. Whole blood specimens were immediately placed on dry ice and stored at −80 Celsius until analysis.

### Primary cell culture

Skeletal muscle tissue was available from five affected individuals for the purpose of primary cell culture. Frozen skeletal muscle was thawed in pre-warmed growth medium (low glucose DMEM, fetal bovine serum, penicillin–streptomycin, gentamycin, and Amphotericin B) and then washed in PBS. Using a scalpel, tissue was finely minced (< 1–2 mm^3^) in 500 *µ*L growth medium. The tissue fragment solution was transferred to a T-25 tissue culture flask (ThermoFisher #169,900) and left to adhere at room temperature for 20 min. Once adhered, 5 mL growth medium was added to the flask and the tissue was incubated for 7 days at 37 ˚C and 5% CO_2_ with a 50% media exchange every 48 h. Once at 90% confluency, primary cultures comprising myoblasts and fibroblasts were split using 5 mL 0.04% trypsin and enriched for CD56^+^ myoblasts by magnetic bead cell sorting per the manufacturer’s instructions (Miltenyi Biotec #130–097-042). The CD56^+^ cell fraction was transferred to a T-25 flask and grown under the same conditions until 90% confluent. Following this, myoblast-enriched primary cultures were split using trypsin, a cell count and viability assay was performed, and a predominantly myogenic cell type confirmed by immunofluorescence microscopy with immunostaining for desmin, a myogenic marker (primary antibody: Thermofisher #PIPA516705) and TE-7, a fibroblast marker (primary antibody: Millipore # CBL-271). The secondary antibody cocktail comprised Alexafluor 488 and 568 (Thermofisher # A-11001 and A-11011, respectively). Primary myoblasts were available from five *RYR1*-RM affected individuals and two otherwise healthy controls. Myoblasts were (a) plated in six T-75 tissue culture flasks in preparation for nicotinamide riboside treatments or (b) plated in XFp microplates for assessment of mitochondrial function according to the manufacturer’s instructions (Agilent # 103,010–100). Once T-75 myoblast cultures were 80% confluent, growth medium was replaced with differentiation medium (high glucose DMEM, horse serum, penicillin–streptomycin, and insulin) to induce formation of multinucleated myotubes. Following five-day treatment with differentiation medium and visual confirmation of multinucleation by light microscopy, nicotinamide riboside treatments were initiated.

### Nicotinamide riboside (NR) treatment

Primary myotube cultures (*n* = 5) were exposed to low or high doses of nicotinamide riboside (0.25 mM or 0.50 mM prepared in dH_2_O; SelleckChem, TX, USA) or vehicle (dH_2_O) for 24 and 72-h. Media was exchanged with each daily administration of nicotinamide riboside or vehicle (differentiation media without nicotinamide riboside). Doses were selected based on the previously reported maximum effective dose for NR in myotubes [17, 18].

### Redox analyses

The following redox analytes were analyzed using a newly developed colorimetric assay shown to be comparable to mass spectrometry: GSH, GSSG, NAD^+^, NADH, and oxidized/reduced form ratios were calculated for each analyte [14]. Analysis of a given redox couple (i.e., ratio of reduced to oxidized forms) captures cellular metabolic balance. Individuals performing redox assays were blinded to study timepoint and treatment allocation. Pelleted primary myotube samples, each comprising approximately 2,000,000 cells, were prepared in proprietary buffer per the manufacturer’s instructions, and stored at −80 Celsius until analysis. Murine skeletal muscle tissue was snap frozen upon collection and stored at −80 Celsius until analysis with results normalized to total protein concentration.

### Oxygen consumption rate (OCR)

Oxygen consumption rate (OCR) in untreated and NR-treated *RYR1-*RM and control primary myotubes was assessed using a XFp extracellular flux analyzer (Seahorse Agilent Technologies., CA, USA). All samples were plated in triplicate at a seeding density of 20,000 cells and grown to 80% confluency before differentiation to myotubes, as described above. Cells were treated with a single low dose (0.25 mM) or high dose (0.50 mM) NR for 24 h prior to analysis in triplicate using the Cell Mito Stress Test, per the manufacturer’s instructions (Agilent Technologies., CA, USA). Results were compared to control (untreated) myotubes derived from the same participants. OCR values were normalized to cell count determined using a Biotek Cytation Cell Imaging Multimode Reader and Hoechst nuclear staining (Biotek, Agilent Technologies., CA, USA).

### Statistics

Descriptive statistics including frequency (percentage) and mean ± standard deviation were generated for all endpoints. Differences in baseline redox analytes and ratios between reduced and oxidized forms (GSH, GSSG, NAD^+^, and NADH), versus otherwise healthy adult controls (*n* = 299) [14], were assessed by one-sample t-tests. Normality of data were assessed by the Shapiro–Wilk test. Group nonparametric comparisons (e.g., *RYR1*-RM versus general population controls, and NAC versus placebo) were made using the Wilcoxon rank sum test. Change over time in redox analytes (Month 6 to Month 12) was assessed by repeated measures analysis of covariance, controlling for age and baseline values. The time and dose-dependent effects of nicotinamide riboside (NR) treatment in *RYR1*-RM primary myotubes were descriptively owing to the limited availability of patient-derived cells. Pearson’s correlation coefficient was used to assess the relationship between age and redox parameters. Statistical analyses were conducted using SAS (Version 9.4) and figures generated using GraphPad Prism (Version 9.0).

## Results

Participant characteristics and genotypes are presented in Tables 1 and S1, respectively. Whole blood specimens were available for a total of 28 trial participants. Pre- and post-treatment specimens were available for 22 participants (NAC and placebo groups: *n* = 12 and *n* = 10, respectively). Six additional participants had only pre-treatment specimen(s) available. Ten participants had specimens available from all study timepoints (month 0, baseline; month 6, pre-treatment; month 12, post-treatment). Skeletal muscle tissue was available from five *RYR1*-RM affected individuals (heterozygous *n* = 3 and compound heterozygous *n* = 2) with four samples suitable for redox analyses and five samples used to generate primary myotube cultures.

**Table 1:** Baseline characteristics of trial participants included in redox analyses

Endpoint	NAC	Placebo	Total Cohort ^a^
	(*n* = 12)	(*n* = 10)	(*n* = 28)
Age, years	33.9 ± 16.2	22.0 ± 16.6	27.4 ± 16.2
Sex (Male), n	5	4	12
Height, cm	158.4 ± 18.2	142.9 ± 16.4	152.6 ± 19.5
Weight, kg	63.3 ± 25.6	42.2 ± 18.3	53.3 ± 25.5
BMI, kg/m^2^	24.3 ± 8.5	19.8 ± 5.5	21.8 ± 7.9
Dominant inheritance, n	10	7	20
Pediatric, n	3	5	10

Data are mean ± standard deviation (SD), or frequency

^a^includes participants with baseline and pre-intervention samples who did not advance to the treatment phase

### Whole blood redox analyses

**Fig. 1:**
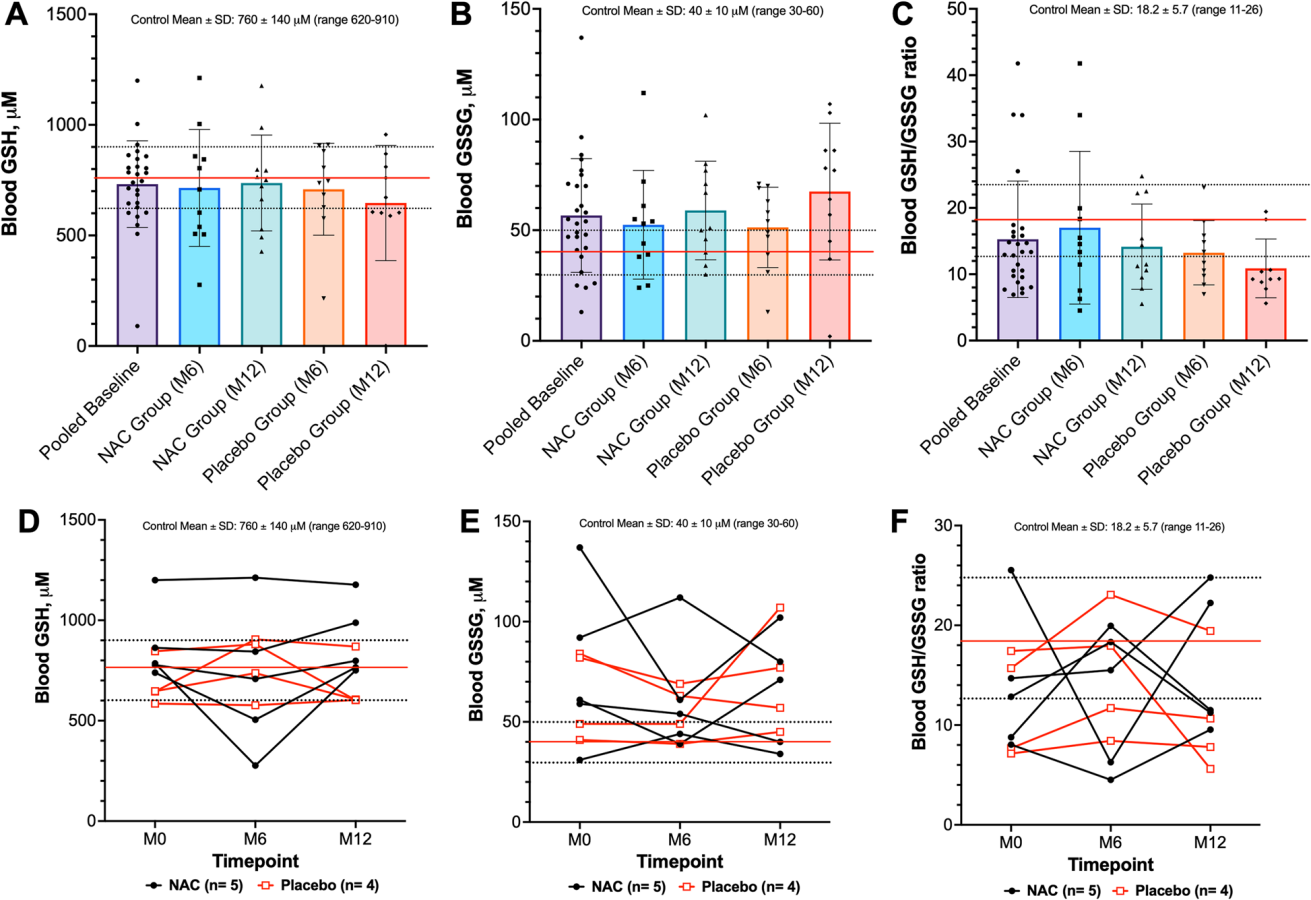
A majority of *RYR1*-RM affected individuals exhibit systemic GSH/GSSG ratios within normal limits that are relatively stable over 12-months. Red and dashed lines represent the mean and standard deviation for the reference population, respectively (*n* = 299). In A-C, bars reflect mean ± SD. **A** In the total cohort (*n* = 27), 78% of participants demonstrated GSH results within normal limits at baseline. In participants assigned to NAC treatment, 5/12 had a GSH concentration below normal limits at baseline. **B** 63% of participants exhibited a GSSG concentration within normal limits at baseline. In participants assigned to NAC treatment, 3/12 had a GSSG concentration above normal limits at baseline. **C** 63% of participants also had a GSH/GSSG ratio within normal limits at baseline. In participants assigned to NAC treatment, 3/12 had a GSH/GSSG ratio below normal limits at baseline. There was no impact of six-month NAC treatment on GSH and GSSG concentrations or GSH/GSSG ratio versus placebo (**D**-**F** and Table 2)

For one participant, GSH and GSSG concentrations were below the limits of detection resulting in a total sample size of *n* = 27. In *RYR1*-RM affected individuals, average baseline GSH concentration was comparable to the general population (732.1 ± 196.0 mM vs. 760.0 ± 140.0 mM, respectively, *p* = 0.47, Fig. 1A) whereas GSSG concentration was higher but within normal limits (56.6 ± 25.7 mM vs. 40.0 ± 10.0 mM, respectively, *p* = 0.002, Fig. 1B). Average baseline GSH/GSSG ratio in *RYR1*-RM affected individuals was comparable to the general population (15.3 ± 8.8 vs. 18.2 ± 5.7, respectively, *p* = 0.095 Fig. 1C). There was no significant change over 12 months in glutathione redox parameters following NAC treatment versus placebo, after controlling for baseline values (Table 2; Fig. 1D-F). Moreover, in participants with data available for this analysis at all three timepoints (NAC *n* = 5, placebo *n* = 4), glutathione redox status was relatively stable over a 12-month period, regardless of treatment allocation. There was no difference in glutathione redox parameters between heterozygous and compound heterozygous *RYR1*-RM and no correlation with participant age (Figure S1 A-F and Table S2).

**Table 2:** Change from baseline in redox parameters

Endpoint	NAC	Placebo	*P*-value^1^
	(*n* = 12)	(*n* = 11)	
GSH, mM	0.022 (−0.124, 0.169)	0.018 (−0.163, 0.199)	0.60
GSSG, mM	0.007 (−0.008, 0.022)	0.016 (−0.003, 0.035)	0.74
GSH/GSSG, ratio	−2.869 (−10.087, 4.350)	−2.357 (−5.410, 0.696)	0.76
NAD^+^, mM	4.007 (−6.149, 14.163)	0.642 (−5.896, 7.180)	0.61
NADH, mM	0.377 (0.051, 0.703)	0.158 (−0.263, 0.579)	0.43
NAD^+^/NADH, ratio	0.815 (−13.464, 15.093)	−1.082 (−10.680, 8.516)	0.85
NADP, mM	−0.218 (−3.019, 2.583)	−2.042 (−4.717, 0.633)	0.30
NADPH, mM	0.688 (0.014, 1.362)	−0.299 (−1.105, 0.507)	0.095
NADP/NADPH, ratio	−23.006 (−72.327, 26.316)	0.982 (−2.611, 4.575)	0.55

Data are mean change (95% Confidence Interval)

^1^From repeated measures ANCOVA, controlling for age and baseline values

**Fig. 2:**
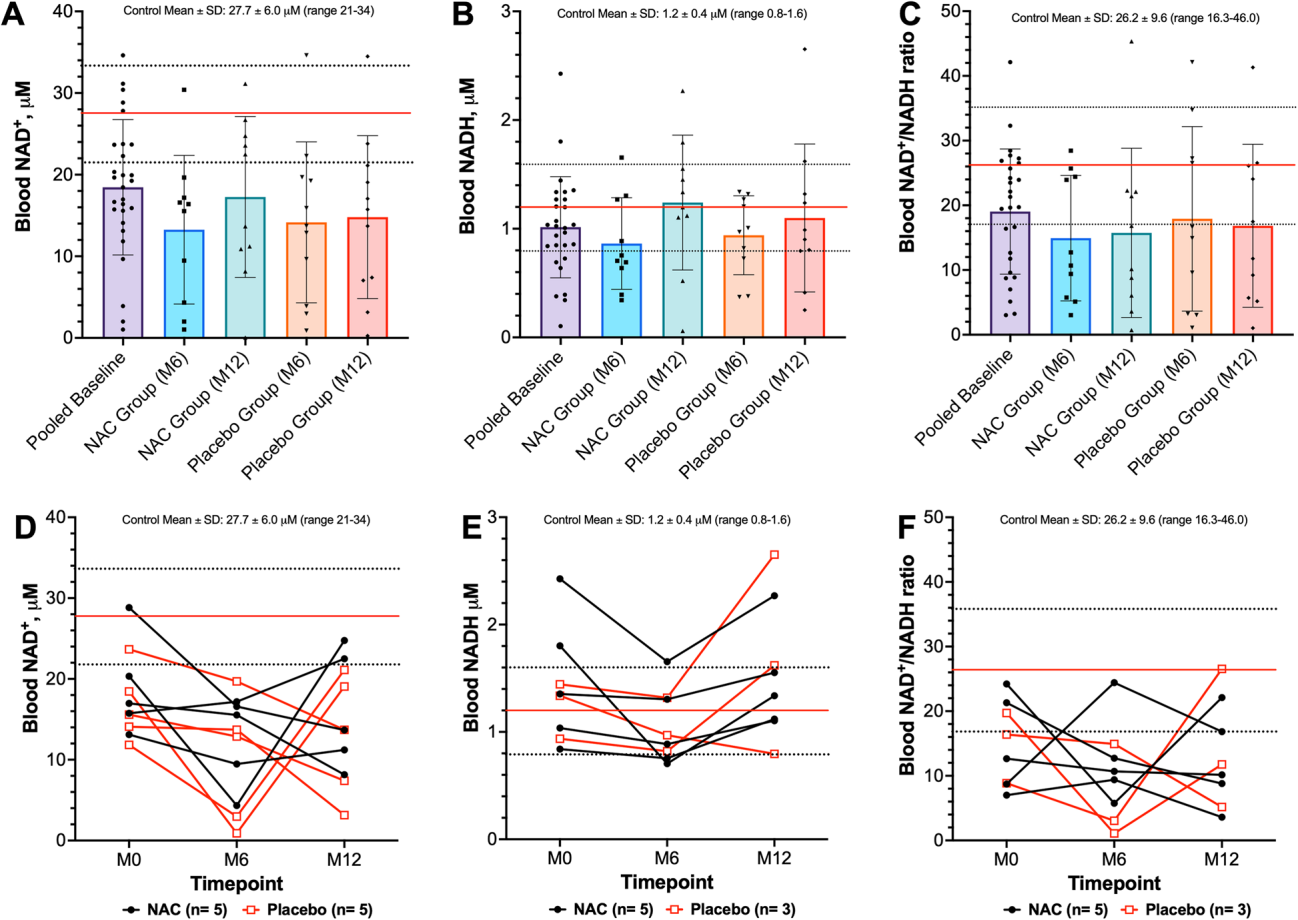
A majority of *RYR1*-RM affected individuals exhibit systemic NAD^+^ deficiency and a decreased NAD^+^/NADH ratio which remain below normal limits over 12-months. Red and dashed lines represent the mean and standard deviation for the reference population, respectively (*n* = 299). In A-C, bars reflect mean ± SD. **A** In the total cohort, 19/28 (68%) of participants demonstrated systemic NAD^**+**^ deficiency at baseline. In participants assigned to NAC treatment, 10/11 (91%) had a NAD^**+**^ concentration below normal limits at baseline. **B** 4/26 (17%) of participants exhibited a diminished NADH concentration at baseline. At baseline, two participants had NADH concentrations above normal limits (ranging from 1.8 to 2.4 µM) and four participants had NADH concentrations below normal limits (ranging from 0.1 to 0.4 µM), Fig. 3B. **C** 18/28(64%) of participants presented with a NAD^+^/NADH ratio within normal limits. In participants assigned to NAC treatment, 6/11 (55%) had a NAD^+^/NADH ratio below normal limits at baseline. There was no impact of six-month NAC treatment on NAD^+^ and NADH concentrations or NAD.^+^/NADH ratio versus placebo, Table 2

A majority of *RYR1*-RM affected individuals 19/28 (68%) exhibited systemic NAD^+^ deficiency (< 21 mM) compared to controls at baseline (18.45 ± 8.29 mM vs. 27.7 ± 6.00 mM, respectively, *p* = < 0.001, Fig. 2A). Average NADH concentration was comparable to the general population (1.01 ± 0.47 mM vs. 1.21 ± 0.39 mM, respectively, *p* = 0.34, Fig. 2B). Of the 19 individuals with systemic NAD^+^ deficiency (14.23 ± 6.05 mM), a subset of nine (*n* = 8 heterozygous, *n* = 1 compound heterozygous) also exhibited a diminished NAD^+^/NADH ratio (7.77 ± 3.46) compared to controls whose values ranged from 16.3 to 46. Nonetheless, the average NAD^+^/NADH ratio in *RYR1*-RM affected individuals was, overall, comparable to the general population (23.7 ± 26.5 vs. 26.2 ± 9.6, *p* = 0.62, respectively). There was no significant change over time in NAD^+^ redox parameters following NAC treatment versus placebo, after controlling for baseline values (Table 2; Fig. 2D-F). For this analysis, in participants with data available at all three timepoints (NAC *n* = 5, placebo *n* = 5), NAD^+^ redox parameters were relatively stable over a 12-month period regardless of treatment allocation, Fig. 2D-F. Aside from an decreased NAD^+^/NADH ratio observed in nine heterozygous participants, there was no difference in NAD^+^ redox parameters based on mode of inheritance (Figures S2 A-C). There was also no correlation between NAD^+^ redox parameters and participant age (Figures S2 D-F).

**Fig. 3:**
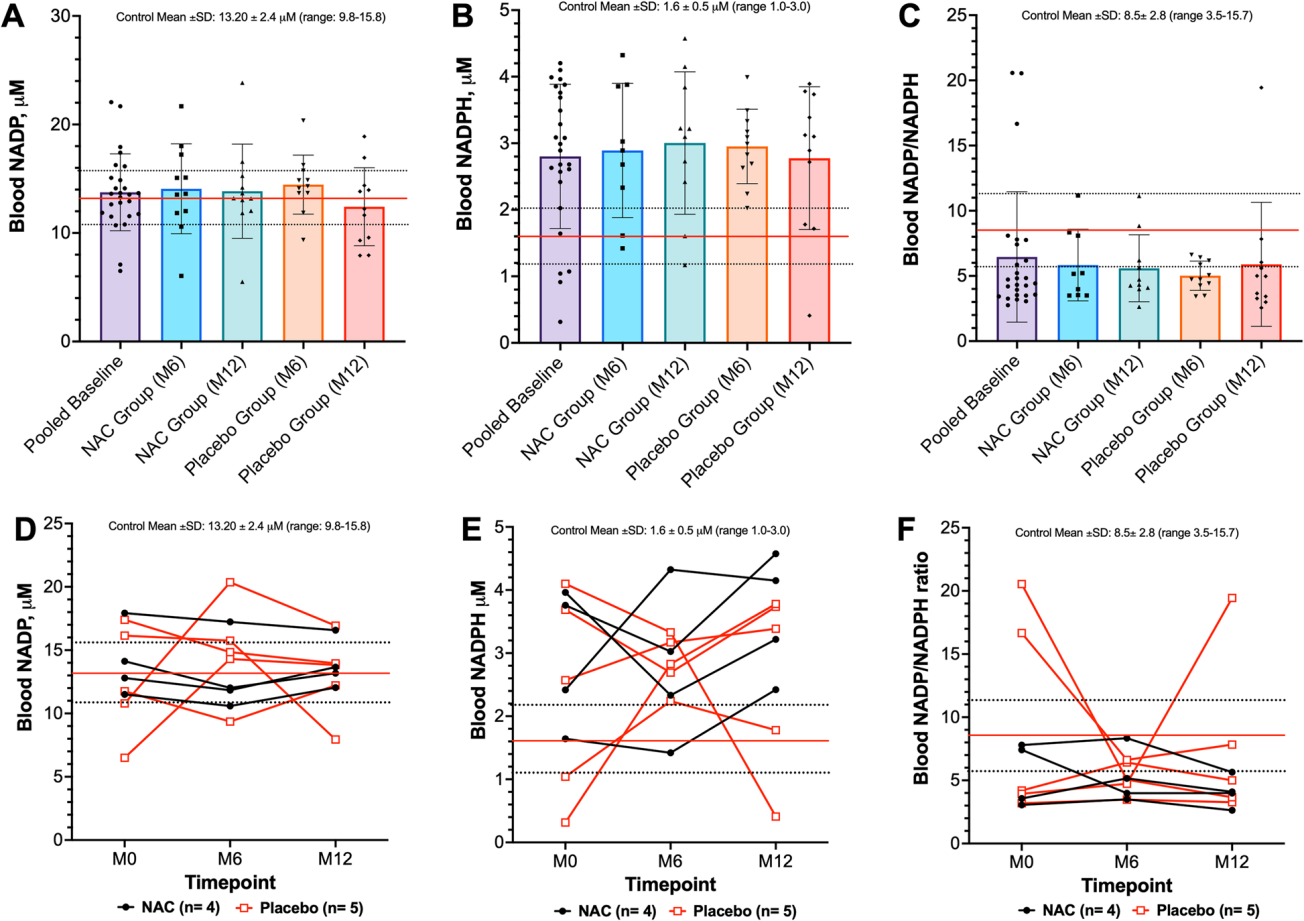
A majority of *RYR1*-RM affected individuals exhibit increased systemic NADPH and a decreased NAD.^+^/NADH ratio which varied in participants over 12-months. Red and dashed lines represent the mean and standard deviation for the reference population, respectively (*n* = 299). In A-C, bars reflect mean ± SD. **A** In the total cohort, 11/26 (42%) of participants had diminished NADP concentrations (< 13.2 mM) at baseline. **B** A majority, 22/26 (85%) of *RYR1*-RM affected individuals demonstrated elevated NADPH concentrations (> 1.6 mM) at baseline. **C** Similarly, a majority 23/26 (88%) of participants had a decreased NADP/NADPH ratio (< 8.5) at baseline. **D**-**F** In participants with data available at all three timepoints (NAC *n* = 4, placebo *n* = 5), NADP redox parameters varied over a 12-month period regardless of treatment allocation. There was no impact of six-month NAC treatment on NADP and NADPH concentrations or NADP/NADPH ratio versus placebo, Table 2

For two participants, NADPH concentrations approached the limit of detection and were deemed outliers, resulting in a total sample size of *n* = 26. Overall, 11/26 (42%) *RYR1*-RM affected individuals had diminished NADP concentrations (< 13.2 mM) at baseline, Fig. 3A. Average NADP concentration was comparable to the general population (13.75 ± 3.54 vs. 13.20 ± 2.4 mM, respectively *p* = 0.28). A majority, 22/26 (85%) of *RYR1*-RM affected individuals demonstrated elevated NADPH concentrations (> 1.6 mM) at baseline, Fig. 3B. Average NADPH concentration was significantly higher compared to the general population (2.80 ± 1.08 vs. 1.6 ± 0.5 mM, respectively *p* = < 0.0001). Similarly, a majority 23/26 (88%) of participants had a decreased NADP/NADPH ratio (< 8.5) at baseline, Fig. 3C. Average NADP/NADPH ratio was significantly lower compared to the general population (6.46 ± 5.00 vs. 8.5 ± 2.8, respectively *p* = 0.0011). For this analysis, in participants with data available at all three timepoints (NAC *n* = 4, placebo *n* = 5), NADP redox parameters varied in a subset of participants over a 12-month period regardless of treatment allocation, (Table 2; Fig. 3D-F). There was no difference in NADP redox parameters based on mode of inheritance (Figures S3 A-C). There was also no correlation between NADP redox parameters and participant age (Figures S3 D-F).

### RYR1-RM skeletal muscle redox analyses

In all skeletal muscle specimens, GSH and GSSG concentrations were below the limit of quantitation. Average NAD^+^ and NADH concentration was significantly higher in *RYR1*-RM skeletal muscle versus controls (Figure S4 A-B) however there was no difference in skeletal muscle NAD^+^/NADH ratio (*p* = 0.31, Figure S4 C). *RYR1*-RM affected individuals demonstrated higher average skeletal muscle NADPH content versus controls, however this difference was not statistically significant (Figure S4 E). There was no difference in NADP or NADP/NADPH ratio between groups (Figure S4 D and F).

### Effects of Nicotinamide Riboside (NR) treatment in RYR1-RM primary myotubes

**Fig. 4:**
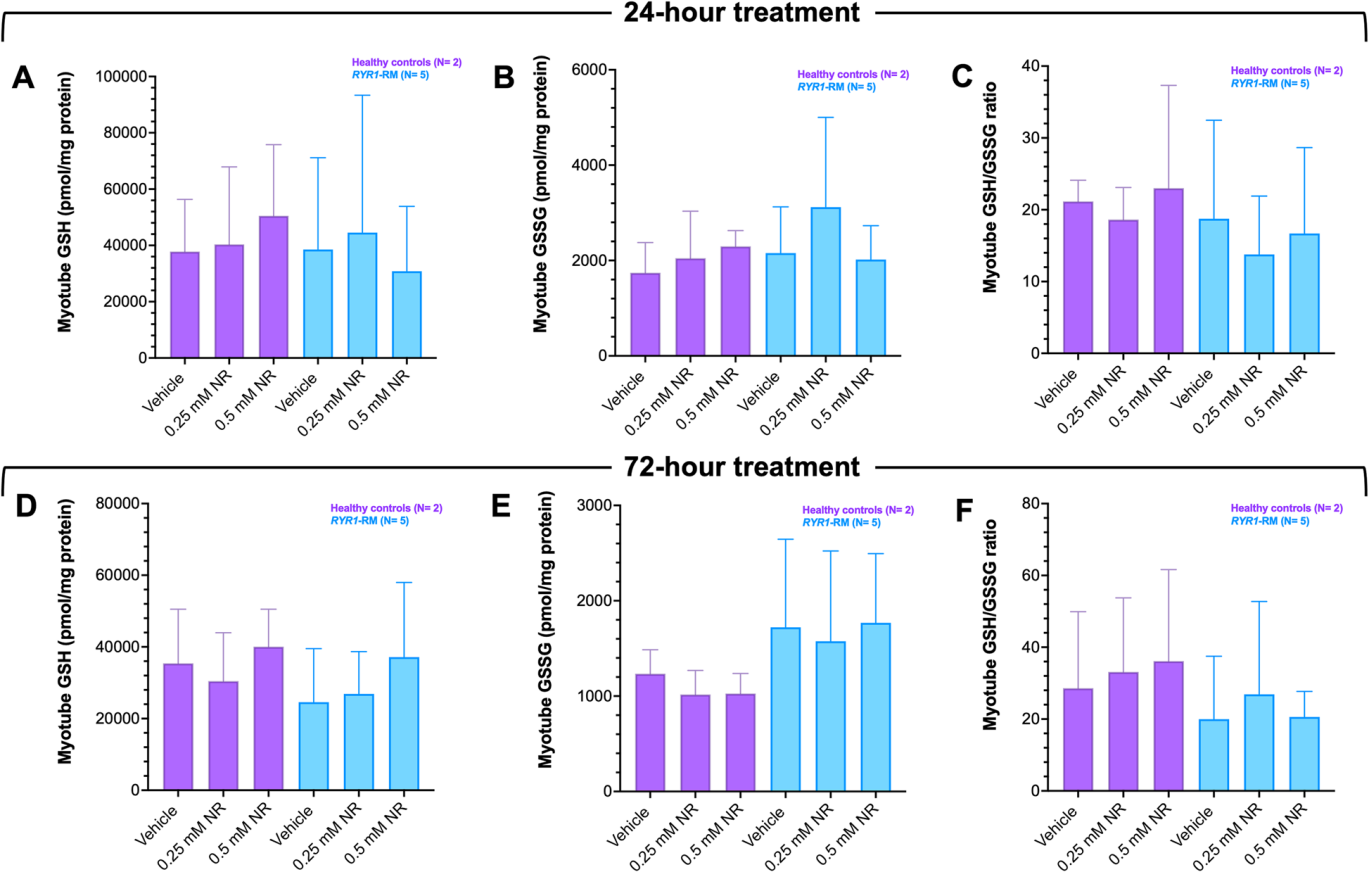
Glutathione redox metabolite concentrations in *RYR1*-RM and control myotube cultures pre- and post-nicotinamide riboside treatment. Myotube cultures were exposed to 0.25 mM or 0.50 mM NR for 24- or 72-h (**A**-**C** and **D**-**F**, respectively). Glutathione redox metabolite concentrations were comparable between vehicle-treated *RYR1*-RM and control cultures Table S2. Glutathione redox metabolite concentrations in both *RYR1*-RM and control cultures were unaffected by NR treatment

**Fig. 5:**
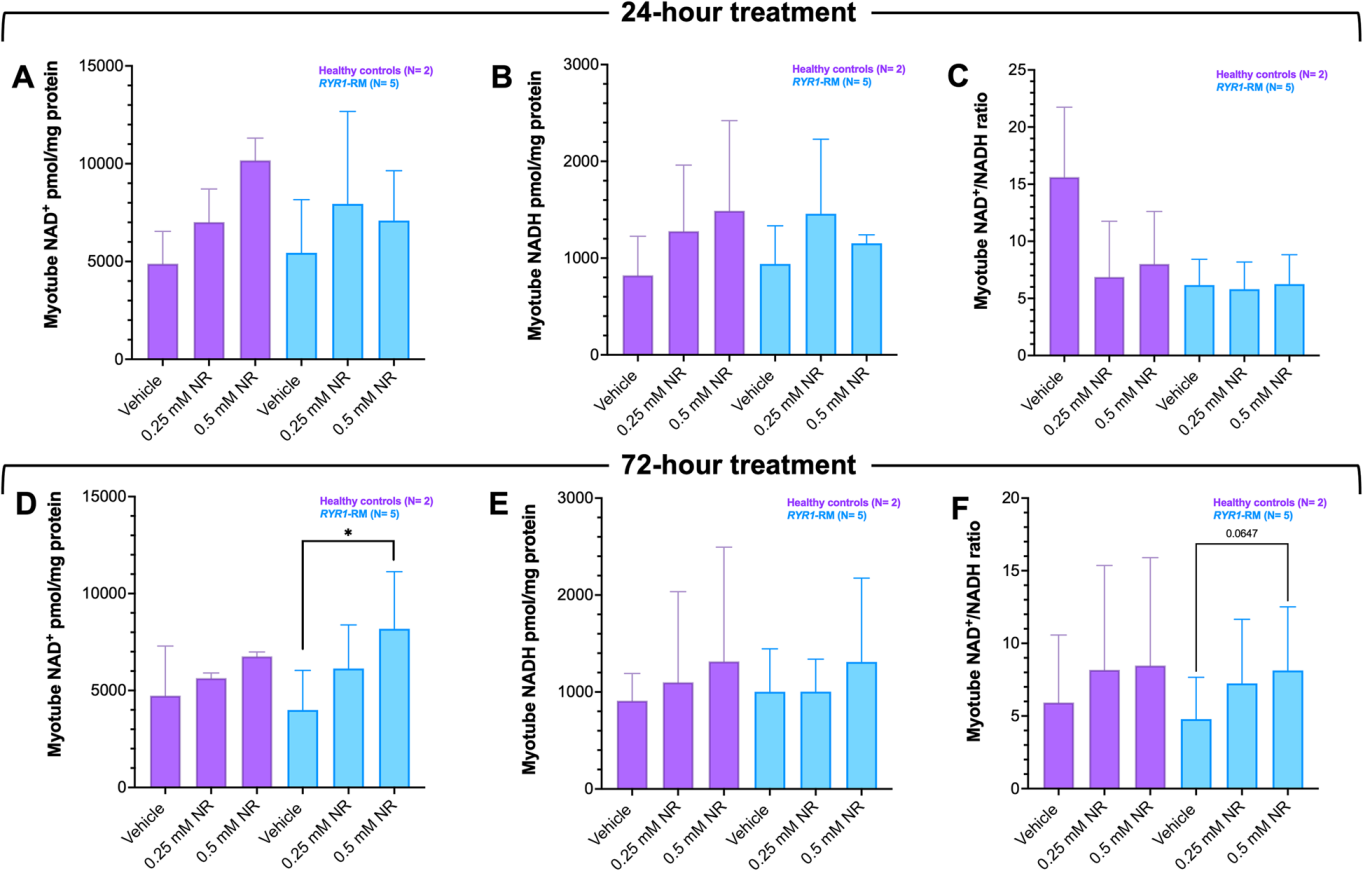
NAD^+^ redox metabolite concentrations in *RYR1*-RM and control myotube cultures pre- and post-nicotinamide riboside (NR) treatment. Data are presented as mean ± SD. Myotube cultures were exposed to 0.25 mM or 0.50 mM NR for 24- or 72-h (**A**-**C** and **D**-**F**, respectively). NAD^+^ redox metabolite concentrations were comparable between vehicle-treated *RYR1*-RM and control cultures, Table S4. **D** 72-h treatment of *RYR1*-RM cultures with NR resulted in a dose and time-dependent increase in NAD^+^ content. **F** This was also reflected in NAD^+^/NADH ratios albeit did not reach statistical significance

**Fig. 6:**
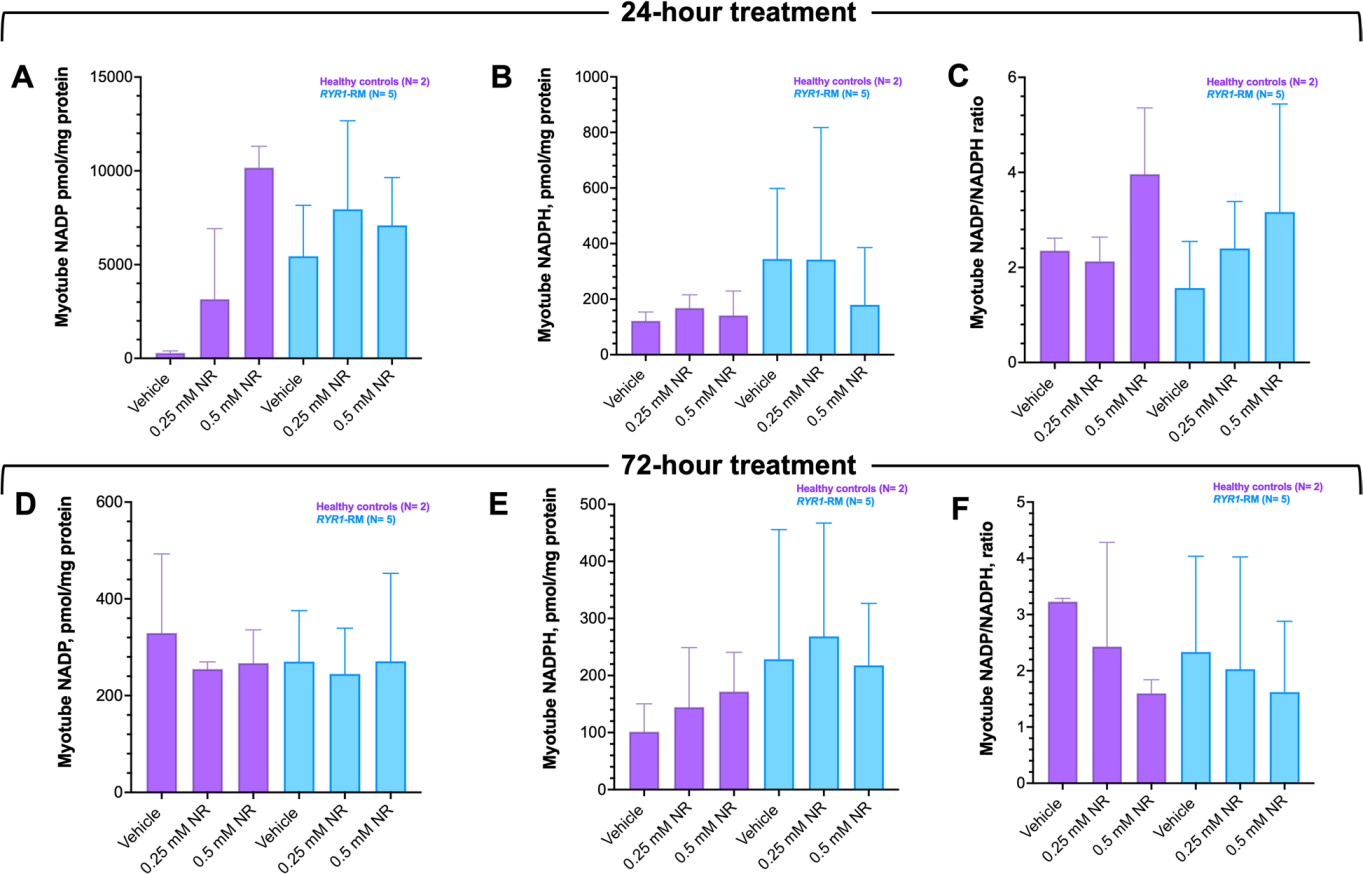
NADP redox metabolite concentrations in *RYR1*-RM and control myotube cultures pre- and post-nicotinamide riboside (NR) treatment. Data are presented as mean ± SD. Myotube cultures were exposed to 0.25 mM or 0.50 mM NR for 24- or 72-h (**A**-**C** and **D**-**F**, respectively). Overall, NADP redox metabolite concentrations were comparable between vehicle-treated *RYR1*-RM and control cultures, Table S5. In *RYR1-*RM cultures, NADP/NADPH ratio increased in a dose dependent manner over 24 h (**C**) however this trend was not observed over a 72-h period (**F**)

In vehicle-treated cultures, there were no significant differences in glutathione redox parameters (GSH, GSSG, GSH/GSSG ratio) between *RYR1*-RM and control myotubes at 24 or 72-h timepoints (Table S3). NR-treatment (0.25 or 0.50 mM) for 24 or 72-h did not modify these parameters, (Fig. 4). In vehicle-treated cultures, there were no significant differences in NAD^+^ redox parameters (NAD^+^, NADH, NAD^+^/NADH ratio) between *RYR1*-RM and control myotubes at 24 or 72-h timepoints (Table S4). NR-treatment resulted in a time and dose-dependent increase in mean ± SD cellular NAD^+^ content in *RYR1*-RM myotubes at 72-h only, Fig. 5. In vehicle-treated cultures, there were no significant differences in NADP redox parameters (NADP, NADPH, NADP/NADPH ratio) between *RYR1*-RM and control myotubes at 24 or 72-h timepoints (Table S5). In NR-treated *RYR1-*RM cultures, NADP/NADPH ratio increased in a dose dependent manner over 24 h however this trend was not observed over a 72-h period, Fig. 6.

There was no significant difference in baseline maximal respiration, ATP production, coupling efficiency, or spare respiratory capacity between *RYR1*-RM and control myotube cultures (Figure S5 A-D). Exposure of myotubes to the highest NR dose (0.50 mM for 24 h) resulted in an apparent dose-dependent increase in maximal respiration and ATP production in *RYR1*-RM cultures (Figure S5 A-B), however formal analyses were precluded by the limited sample size. NR treatment did not significantly impact coupling efficiency or spare respiratory capacity at the doses tested in *RYR1*-RM or control cultures (Figure S5 C-D). Mean ± SD basal oxygen consumption rate was higher in *RYR1*-RM cultures compared to otherwise healthy (52.60 ± 27.71 versus 21.78 ± 6.83, respectively).

### Redox state in Ryr1 Y524S skeletal muscle and whole blood

In 12-month-old *Ryr1* Y524S mice, there were no significant differences observed in individual redox analyte concentrations or ratios in soleus muscle tissue (Figure S6) or whole blood specimens (Figure S7).

## Discussion

Redox imbalance resulting in excessive oxidative stress has been implicated in many neuromuscular disorders [19], including in *RYR1* disease model systems and patients with *RYR1*-RM [8, 10, 15, 20]. Our analysis of *RYR1*-RM banked whole blood specimens utilized a substantially larger control dataset than previously reported (*n* = 299 versus *n* = 24). In humans, primary biosynthesis of NAD^+^ is via the salvage pathway and the important roles of this metabolite in cellular energy metabolism and hydrogen ion transfer in redox reactions, are well established [21, 22].

Here, we provide preliminary evidence that systemic NAD^+^ deficiency is present and relatively stable over a 12-month period in a subset of *RYR1*-RM affected individuals, and was unaffected by a six-month course of oral NAC. Although NAD^+^/NADH ratio was comparable to the general population overall, the significant NAD^+^ depletion may in itself represent added burden on the redox system. Additional preliminary evidence suggests that systemic NADPH concentrations are higher and NADP/NADPH ratios are lower in a subset of *RYR1*-RM affected individuals when compared to general population norms.

Prior nonclinical and clinical studies in a range of neurological disorders have reported NAD^+^ deficiency that is responsive to treatment with NAD^+^ precursor compounds, such as niacin and nicotinamide riboside (NR) [12, 13, 23]. NR can cross the cell membrane and is preferentially utilized by skeletal muscle cells for salvage pathway-driven NAD^+^ biosynthesis, and has favorable pharmacokinetic and pharmacodynamic properties compared to other precursor compounds such as nicotinic acid (NA), nicotinamide (NAM), and nicotinamide mononucleotide (NMN) [24, 25]. In the myocyte, conversion of NR to NAD^+^ is facilitated by nicotinamide riboside kinase 2 (NMRK2) which is principally expressed in skeletal muscle [18]. In RyR native membrane experiments, which do not fully recapitulate the in vivo environment, NAD^+^ has been reported to directly bind to RyR1 (N-terminal domain) and result in increased channel activity [26]. However NAD^+^-mediated RyR1 activation is likely mitigated by competitive binding of physiologic levels of ATP in vivo [27]. In the context of *RYR1*-RM, it is possible that disrupted redox balance, due to excessive mitochondrial calcium uptake, the associated oxidative stress, and further dysfunction of the ryanodine receptor due to post-translational modifications of oxidant-sensitive residues could place additive burden on redox homeostasis, resulting in disrupted NAD^+^ and NADP levels. Additional mechanistic studies are necessary to investigate this hypothesis further. Nonetheless, in this study, NR increased cellular NAD^+^ concentrations in a dose and time-dependent manner and appeared to favorably modify mitochondrial oxygen consumption rate and ATP production in *RYR1*-RM primary myotubes. In skeletal muscle, NAD^+^ synthesis and breakdown are tightly regulated [28]. In the limited number of available patient-derived tibialis anterior skeletal muscle samples (*n* = 4), NAD^+^ and NADH concentrations were higher compared to controls, however NAD^+^/NADH ratios were comparable indicating maintenance of this NAD^+^ redox homeostasis at the tissue level. Nonetheless, further work is necessary to replicate this observation and also to determine (a) the relative NAD^+^ turnover rate/flux in *RYR1*-RM skeletal muscle mitochondrial and cytosolic compartments and (b) if the observed NAD^+^/NADH redox balance holds true when tested in patient-derived slow-twitch/oxidative skeletal muscle samples (e.g., soleus), since these have a higher mitochondrial content and thus greater potential for perturbed redox homeostasis. In adults with mitochondrial myopathy and NAD^+^ deficiency, niacin treatment restored systemic and skeletal muscle tissue NAD^+^ concentrations and resulted in improvement muscle strength and performance [13]. Collectively, these results warrant prospective exploration of systemic NAD^+^ deficiency in *RYR1*-RM and, if replicated, could support a potential therapeutic approach of NAD^+^ repletion in those with baseline deficiency. Additional research in a larger cohort which includes more severely affected individuals is necessary to both replicate and broaden the applicability of these findings.

A diminished systemic NADP/NADPH ratio has been reported in various disease states, including cancer, Parkinson’s, and Alzheimer’s disease [14]. Most *RYR1*-RM affected individuals also had an increased systemic NADPH concentration (85%) and decreased NADP/NADPH ratio (88%) when compared to general population norms. Elevated NADPH is associated with a pro-oxidant state owing to its role as a substrate for NADPH oxidase (NOX)- reactive oxygen species generation (hydrogen peroxide and superoxide) [21] which has been identified as a component of disease pathogenesis in several *Ryr1* murine model systems [7, 29], likely owing to intracellular calcium dysregulation that perpetuates RyR1 dysfunction through aberrant thiol oxidation [30].

Although other pharmacologic therapeutic approaches are in development for *RYR1*-RM, these are focused on addressing the primary pathomechanism rather than downstream sequalae such as redox imbalance. For example, Rycal molecule ARM210, which targets RyR1-mediated sarcoplasmic reticulum calcium leak by stabilizing the RyR1 closed state, has been tested in a phase 1 open-label trial of adults with *RYR1*-RM [31] whereas HDAC and methyltransferase inhibitors are being investigated in compound heterozygous murine model systems with the goal of promoting increased RyR1 protein expression in compound heterozygous *RYR1*-RM affected individuals who harbor at least one loss of function variant [32]. It is unclear if the above approaches will fully address the downstream disruption of redox balance resulting from intracellular calcium dysregulation, which has been observed across genotypes [10]. Indeed, a combinatorial therapeutic approach is foreseeable for *RYR1*-RM given the significant disease heterogeneity. A parallel investigation of adjunctive approaches to support restoration of redox balance, such as NAD^+^ repletion, is therefore warranted. NR is commercially available and is considered generally regarded as safe (GRAS) by the U.S. Food and Drug Administration (FDA) [33], has an established human pharmacokinetic profile [33, 34], and could thus be readily investigated in a phase I/II clinical trial given the precedent in other neurological disorders (NCT05590468, NCT03568968, NCT05617508, NCT05740722). Careful consideration should be placed on the stratification and eligibility criteria for such a study, preferably with enrollment of *RYR1*-RM affected individuals who have clear evidence of baseline systemic NAD^+^ deficiency (i.e. potential responders).

In our reanalysis of systemic glutathione redox status of *RYR1*-RM affected individuals, GSH/GSSG ratios were comparable to otherwise healthy controls, deviating from prior reports [10, 35] and were also relatively stable over 12 months, irrespective of NAC treatment. NR treatment of primary myotubes did not modify GSH/GSSG ratio in *RYR1*-RM or control cultures, which was an expected finding given that NR does not participate in glutathione synthesis. Similarly, our *Ryr1* Y524S analyses deviated from prior reports [15], as no evidence of glutathione redox imbalance was observed systemically or in skeletal muscle. These differences may be explained by nuances in assay methodology such as the extraction of glutathione under acidic conditions. The redox analytical methodology used here does not utilize acids for metabolite extraction thus limiting artefactual conversion of GSH to GSSG during sample preparation [36]. Adoption of standardized methods for redox analyses across rare disease studies will help ensure data can be reliably compared. Furthermore, additional work is needed to investigate redox balance in more clinically relevant *RYR1-*RM model systems that exhibit myopathic features [32, 37, 38].

Our findings are limited by the retrospective design which resulted in a small number of samples available for in vitro NR experiments, precluding firm conclusions from subgroup analyses. Samples were analyzed following extended storage without freeze–thaw cycles. These conditions are not expected to impact the results based on analyte stability studies [14]. Currently, pediatric samples are not available within the control dataset however work to address this is ongoing. Given stringent NAC trial eligibility criteria, future studies should aim to expand NAD^+^ and NADP redox analyses to the broader *RYR1-*RM disease spectrum, such as more severely affected individuals and those with less common phenotypes. Finally, the degree of NAD^+^ and NADP redox imbalance was variable and not observed in all samples, further reiterating the heterogeneity of *RYR1*-RM.

In conclusion, we provide preliminary evidence that systemic NAD^+^ deficiency and increased systemic NADP levels are present in a subset of *RYR1*-RM affected individuals. Furthermore, patient-derived myotubes responded to NR but only at the 72-h testing interval. Further experiments are warranted to confirm these observations, ideally utilizing a larger sample size. Prospective longitudinal studies are planned to further evaluate NAD^+^ and NADP redox dyshomeostasis in *RYR1*-RM and the potential utility of NAD^+^ repletion in this population.

## Supplementary Information

Supplementary Material 1

## Data Availability

The datasets used and/or analyzed during the current study are available from the corresponding author on reasonable request.

## Abbreviations

ATP: Adenosine triphosphate
DMEM: Dulbecco's Modified Eagle Medium
DMSO: Dimethyl sulfoxide
FDA: U.S. Food and Drug Administration
GRAS: Generally recognized as safe
GSH: Glutathione
GSSG: Glutathione disulfide
HDAC: Histone deacetylase
NA: Nicotinic acid
NAC: *N-*Acetylcysteine
NAD^+^: Nicotinamide adenine dinucleotide
NADH: Nicotinamide adenine dinucleotide plus hydrogen
NAM: Nicotinamide
NDRI: National Disease Research Interchange
NIH: National Institutes of Health
NMN: Nicotinamide mononucleotide
NMRK2: Nicotinamide riboside kinase 2
NR: Nicotinamide riboside
OCR: Oxygen consumption rate
*RYR1*-RD: *RYR1*-Related disorders
*RYR1*-RM: *RYR1*-Related myopathy
SR: Sarcoplasmic reticulum
