# Supplementary material for: NAD+ dyshomeostasis in RYR1-related myopathies
Source: Skelet Muscle. 2025 Aug 22;15:22. doi: 10.1186/s13395-025-00390-6 (PMC12374369; doi:10.1186/s13395-025-00390-6)
Supplement: Supplementary file 1 — Supplementary Material 1 [file 13395_2025_390_MOESM1_ESM.docx]

**Supplementary Appendix**

**NAD^+^ dyshomeostasis in *RYR1*‑related Myopathies**

Tokunbor A. Lawal, Ph.D.^1,2^, Willa Riekhof B.S.^1^, Linda Groom^3^, Pooja Varma B.S.^1^, Irene C. Chrismer BSN^1,2^, Angela Kokkinis R.N.^4^, Christopher Grunseich M.D.^4^, Jessica W. Witherspoon, Ph.D.^1^, Muslima S. Razaqyar, B.A.^1^, Ninet Sinaii, Ph.D., M.P.H.^5^, Katherine G. Meilleur, Ph.D.^1^, Lichen Xiang, Ph.D.^1^, Jana Buzkova, Ph.D.^6^, Liliya Euro, Ph.D.^6^, Payam Mohassel, M.D.,^7,8^, Robert T. Dirksen, Ph.D.^3^, Joshua J. Todd, Ph.D.^8,9^.

**Author affiliations:**

^1^ Skeletal Myopathies Unit, Translational Biobehavioral and Health Disparities Branch, NIH Clinical Center, National Institutes of Health, Bethesda, MD, USA.

^2^ Muscle Disease Unit, National Institute of Nursing Research, National Institutes of Health, Bethesda, MD, USA.

^3^ Department of Pharmacology and Physiology, University of Rochester Medical Center, Rochester, NY, USA.

^4^ Inherited Neuromuscular Diseases Unit, National Institute of Neurological Disorders and Stroke, National Institutes of Health, Bethesda, MD, USA.

^5^ Biostatistics and Clinical Epidemiology Service, National Institutes of Health Clinical Center, Bethesda, MD, USA.

^6^ NADMed Ltd. Biomedicum 1, Haartmaninkatu 8, Helsinki, Finland.

^7^ Johns Hopkins University School of Medicine, Baltimore, MD.

^8^ Neuromuscular and Neurogenetic Disorders of Childhood Section, National Institute of Neurological Disorders and Stroke, National Institutes of Health, Bethesda, MD, USA.

^9^ Clinical Trials Unit, National Institute of Neurological Disorders and Stroke, Bethesda, MD, USA.

Correspondence to: Joshua J. Todd, Ph.D. National Institute of Neurological Disorders and Stroke, National Institutes of Health, Bethesda, MD, USA, 20814. [joshua.todd@nih.gov](mailto:joshua.todd@nih.gov)

Table of Contents

[Supplementary Tables 3](#_Toc196296346)

[Table S1.](#_Toc196296347) *[RYR1](#_Toc196296347)* [genotypes reflected in the analysis cohort by sample type 3](#_Toc196296347)

[Table S2. Redox analyte levels by age group Error! Bookmark not defined.](#_Toc196296348)

[Table S3. Mean ± SD Glutathione Redox Metabolite Concentrations in Primary Myotube 24 and 72hr NR Experiments 6](#_Toc196296349)

[Table S4. Mean ± SD NAD](#_Toc196296350)^[+](#_Toc196296350)^ [Redox Metabolite Concentrations in Primary Myotube 24 and 72hr NR Experiments 6](#_Toc196296350)

[Table S5. Mean ± SD NADP](#_Toc196296351)[Redox Metabolite Concentrations in Primary Myotube 24 and 72hr NR Experiments 7](#_Toc196296351)

[Supplementary Figures 9](#_Toc196296352)

[Figure S1. Blood glutathione redox parameters evaluated by](#_Toc196296353) *[RYR1](#_Toc196296353)*[-RM mode of inheritance and participant age at time of baseline sample collection. 9](#_Toc196296353)

[Figure S2. Blood NAD](#_Toc196296354)^[+](#_Toc196296354)^ [redox parameters evaluated by](#_Toc196296354) *[RYR1](#_Toc196296354)*[-RM mode of inheritance and participant age at time of baseline sample collection. 10](#_Toc196296354)

[Figure S3. Blood NADP](#_Toc196296355)[redox parameters evaluated by](#_Toc196296355) *[RYR1](#_Toc196296355)*[-RM mode of inheritance and participant age at time of baseline sample collection. 11](#_Toc196296355)

[Figure S4. NAD](#_Toc196296356)^[+](#_Toc196296356)^ [redox metabolite concentrations in](#_Toc196296356) *[RYR1](#_Toc196296356)*[-RM and control skeletal muscle tissue. 12](#_Toc196296356)

[Figure S5. Mitochondrial function in](#_Toc196296357) *[RYR1](#_Toc196296357)*[-RM and control myotubes pre- and post-nicotinamide riboside (NR) treatment. 13](#_Toc196296357)

[Figure S6. Glutathione, NAD](#_Toc196296358)^[+](#_Toc196296358)^[, and NADP redox metabolite concentrations and ratios in](#_Toc196296358) *[Ryr1](#_Toc196296358)* [Y524S soleus muscle. 14](#_Toc196296358)

[Figure S7. Glutathione, NAD](#_Toc196296359)^[+](#_Toc196296359)^[, and NADP redox metabolite concentrations and ratios in](#_Toc196296359) *[Ryr1](#_Toc196296359)* [Y524S whole blood. 15](#_Toc196296359)

# **Supplementary Tables**

| **Table S1. *RYR1* genotypes reflected in the analysis cohort by sample type** | | |
| --- | --- | --- |
| ***Blood*** | ***Nucleotide change*** | ***Amino acid change*** |
| Heterozygous cases | c.14763C>G | p.Phe4921Leu |
|  | c.7354C>T | p.Arg2452Trp |
|  | c.14818G>A | p.Ala4940Thr |
|  | c.14458G>A | p.Gly4820Arg |
|  | c.14458G>A | p.Gly4820Arg |
|  | c.14458G>A | p.Gly4820Arg |
|  | c.14582G>A | p.Arg4861His |
|  | c.14582G>A | p.Arg4861His |
|  | c.14681C>A | p.Ala4894Asp |
|  | c.14807T>G | p.Leu4936Arg |
|  | c.12083C>T | p.Ser4028Leu |
|  | c.14422_14423delTTinsAA | p.Phe4808Asn |
|  | c.14558C>T | p.Thr4853Ile |
|  | c.14731G>A | p.Glu4911Lys |
|  | c.13513G>C | p.Asp4505His |
|  | c.13513G>C | p.Asp4505His |
|  | c.14681C>A | p.Ala4894Asp |
|  | c.14818G>A | p.Ala4940Thr |
|  | c.13513G>C | p.Asp4505His |
| Compound heterozygous cases | c.6721C>T c.325C>T c.2122G>A c.1453A>G | p.Arg2241* p.Arg109Trp p.Asp708Asn p.Met485Val |
|  | c.14582G>A c.13331_13351dup | p.Arg4861His p.Gly4444-Gly4450dup |
|  | c.14210G>A c.12063_12064dupCA c.6797-9C>T | p.Arg4737Gln p.Met4022Thrfs*4 N/A (intronic) |
|  | c.4485_4500del16 c.7060_7062delGTG | p.W1495* p.V2354del |
|  | c.6671G>A c.14818G>A | p.Arg2224His p.Ala4940Thr |
|  | c.7300G>A c.14623A>G | p.Gly2434Arg p.Met4875Val |
|  | c.14126C>T c.6721C>T | p.Thr4709Met p.Arg2241* |
|  | c.10097G>A c.4711A>G c. 11798A>G c.14731G>A | p.Arg3366His p.Ile1571Val p.Tyr3933Cys p.Glu4911Lys |
|  | c.838C>T c.9716T>A | p.Arg280* p.Met3239Lys |
| ***Skeletal muscle*** | ***Nucleotide change*** | ***Amino acid change*** |
| Heterozygous cases | c.14681C>A | p.Ala4894Asp |
|  | c.7354C>T | p.Arg2452Trp |
|  | c.14731G>A | p.Glu4911Lys |
| Compound heterozygous cases | c.6721C>T c.325C>T c.2122G>A c.1453A>G | p.Arg2241* p.Arg109Trp p.Asp708Asn p.Met485Val |
| ***Primary myotubes*** | ***Nucleotide change*** | ***Amino acid change*** |
| Heterozygous cases | c.2923C>T | p.Arg975Trp |
|  | c.7354C>T | p.Arg2452Trp |
|  | c.14731G>A | p.Glu4911Lys |
| Compound heterozygous cases | c.6721C>T c.325C>T c.2122G>A c.1453A>G | p.Arg2241* p.Arg109Trp p.Asp708Asn p.Met485Val |
|  | c.5140_512del c.14126C>T c.4999C>T | p.Leu1714del p.Thr4709Met p.Arg1667Cys |

| **Table S2. Redox analyte levels by age group and mode of inheritance** | | | | |
| --- | --- | --- | --- | --- |
| **Group** | **Dominant inheritance (n)** | **GSH (µM)** | **GSSG (µM)** | **GSH/GSSG** |
| Adult (n= 17) | 13 | 720 ± 210 | 50 ± 20 | 16.8 ± 10.7 |
| Pediatric (n= 10) | 6 | 760 ± 190 | 60 ± 30 | 12.8 ± 2.9 |
|  |  | **NAD+ (µM)** | **NADH (µM)** | **NAD+/NADH** |
| Adult (n= 18) | 14 | 20.6 ±7.7 | 1.1 ± 0.5 | 20.0 ± 8.3 |
| Pediatric (n= 9) | 6 | 14.4 ± 8.7 | 0.9 ± 0.3 | 17.0 ± 12.3 |
|  |  | **NADP+ (µM)** | **NADPH (µM)** | **NADP+/NADPH** |
| Adult (n= 17) | 13 | 13.6 ± 4.1 | 2.6 ± 1.3 | 7.4 ± 6.0 |
| Pediatric (n= 9) | 6 | 14.1 ± 2.3 | 3.1 ± 0.6 | 4.7 ± 1.3 |

| **Table S3. Mean ± SD Glutathione Redox Metabolite Concentrations in Primary Myotube 24 and 72hr NR Experiments** | | | |  |
| --- | --- | --- | --- | --- |
| **Metabolite** | **Treatment** | **Average Cellular Metabolite Concentration** | |  |
|  |  | *RYR1*-RM Myotubes (n= 5) | Control Myotubes (n=2) | P-value^1^ |
| ***24-hour experiments*** | | | |  |
| GSH, pmol/mg protein | Vehicle | 38529 ± 32603 | 37795 ± 18562 | 0.98 |
|  | 0.25 mM NR | 44527 ± 48834 | 40359 ± 27536 | 0.89 |
|  | 0.50 mM NR | 30803 ± 23048 | 50506 ± 2525 | 0.50 |
|  | | | |  |
| GSSG, pmol/mg protein | Vehicle | 2155 ± 971 | 1742 ± 635 | 0.69 |
|  | 0.25 mM NR | 3119 ± 1883 | 2048 ± 986 | 0.30 |
|  | 0.50 mM NR | 2021 ± 709 | 2298 ± 331 | 0.79 |
|  | | | |  |
| GSH:GSSG, ratio | Vehicle | 18.75 ± 13.70 | 21.17 ± 2.95 | 0.80 |
|  | 0.25 mM NR | 13.77 ± 8.13 | 18.63 ± 4.48 | 0.61 |
|  | 0.50 mM NR | 16.70 ± 11.95 | 23.01 ± 14.30 | 0.50 |
| ***72-hour experiments*** | | | |  |
| GSH, pmol/mg protein | Vehicle | 24565 ± 14939 | 32596 ± 19088 | 0.57 |
|  | 0.25 mM NR | 26876 ± 11809 | 31078 ± 12607 | 0.76 |
|  | 0.50 mM NR | 37135 ± 20852 | 34391 ± 18491 | 0.84 |
|  | | | |  |
| GSSG, pmol/mg protein | Vehicle | 1721 ± 923.5 | 1234 ± 251.9 | 0.47 |
|  | 0.25 mM NR | 1575 ± 946.6 | 1017 ± 252.8 | 0.41 |
|  | 0.50 mM NR | 1768 ± 726.6 | 1026 ± 211.7 | 0.28 |
|  | | | |  |
| GSH:GSSG, ratio | Vehicle | 19.96 ± 17.5 | 28.59 ± 21.3 | 0.60 |
|  | 0.25 mM NR | 26.86 ± 25.9 | 33.13 ± 20.6 | 0.70 |
|  | 0.50 mM NR | 20.60 ± 7.1 | 36.15 ± 25.5 | 0.35 |
| ^1^ Repeated measures analyses using mixed effects models, unadjusted p-values. Analyses were performed between groups (i.e., *RYR1*-RM versus control myotubes). | | | | |

| **Table S4. Mean ± SD NAD^+^ Redox Metabolite Concentrations in Primary Myotube 24 and 72hr NR Experiments** | | | |  |
| --- | --- | --- | --- | --- |
| **Metabolite** | **Treatment** | **Average Cellular Metabolite Concentration** | |  |
|  |  | *RYR1*-RM Myotubes (n= 5) | Control Myotubes (n= 2) | P-value^1^ |
| ***24-hour experiments*** | | | |  |
| NAD^+^, pmol/mg protein | Vehicle | 5443 ± 2719 | 4891 ± 1650 | 0.84 |
|  | 0.25 mM NR | 7945 ± 4726 | 7016 ± 1693 | 0.73 |
|  | 0.50 mM NR | 7090 ± 2550 | 10174 ± 1136 | 0.26 |
|  | | | |  |
| NADH, pmol/mg protein | Vehicle | 939.7 ± 394.5 | 361.3 ± 247.0 | 0.23 |
|  | 0.25 mM NR | 1458 ± 771.2 | 1246 ± 638.4 | 0.65 |
|  | 0.50 mM NR | 1153 ± 88.0 | 1567 ± 1040 | 0.39 |
|  | | | |  |
| NAD^+^:NADH, ratio | Vehicle | 6.2 ± 2.3 | 15.6 ± 6.1 | 0.043* |
|  | 0.25 mM NR | 5.8 ± 2.4 | 6.9 ± 4.9 | 0.69 |
|  | 0.50 mM NR | 6.3 ± 2.6 | 8.0 ± 4.6 | 0.52 |
| ***72-hour experiments*** | | | |  |
| NAD^+^, pmol/mg protein | Vehicle | 3993 ± 2045 | 4732 ± 2561 | 0.70 |
|  | 0.25 mM NR | 6130 ± 2252 | 5636 ± 2667 | 0.80 |
|  | 0.50 mM NR | 8183 ± 2943 | 6761 ± 226 | 0.47 |
|  | | | |  |
| NADH, pmol/mg protein | Vehicle | 1002 ± 442.8 | 909.5 ± 281.1 | 0.87 |
|  | 0.25 mM NR | 1003 ± 334.7 | 1100 ± 934.7 | 0.86 |
|  | 0.50 mM NR | 1309 ± 864.8 | 1315 ± 1179 | 0.99 |
|  | | | |  |
| NAD^+^:NADH, ratio | Vehicle | 4.8 ± 2.9 | 5.9 ± 5.0 | 0.77 |
|  | 0.25 mM NR | 7.2 ± 4.4 | 8.2 ± 7.2 | 0.81 |
|  | 0.50 mM NR | 8.1 ± 4.4 | 8.5 ± 7.4 | 0.93 |
| ^1^ Repeated measures analyses using mixed effects models, unadjusted p-values. Analyses were performed between groups (i.e., *RYR1*-RM versus control myotubes).  *Adjusted p-value | | | | |

| **Table S5. Mean ± SD NADP Redox Metabolite Concentrations in Primary Myotube 24 and 72hr NR Experiments** | | | |  |
| --- | --- | --- | --- | --- |
| **Metabolite** | **Treatment** | **Average Cellular Metabolite Concentration** | |  |
|  |  | *RYR1*-RM Myotubes (n= 5) | Control Myotubes (n= 2) | P-value^1^ |
| ***24-hour experiments*** | | | |  |
| NADP, pmol/mg protein | Vehicle | 422.7 ± 450.4 | 291.5 ± 106.5 | 0.71 |
|  | 0.25 mM NR | 545.3 ±  | 371.0 ±  | 0.58 |
|  | 0.50 mM NR | 306.8 ± 183.6 | 500.7  | 0.62 |
|  | | | |  |
| NADPH, pmol/mg protein | Vehicle | 344.4 ± 254.3 | 122.0 ± 31.8 | 0.39 |
|  | 0.25 mM NR | 342.2 ± 4 | 168.2 ± 47.3 | 0.88 |
|  | 0.50 mM NR | 179.2 ± 206.2 | 141.6 ±  | 0.50 |
|  | | | |  |
| NADP:NADPH, ratio | Vehicle | 1.6 ± 1.0 | 2.4 ± 0.3 | 0.52 |
|  | 0.25 mM NR | 2.4 ± 1.0 | 2.1 ± 0.5 | 0.83 |
|  | 0.50 mM NR | 3.2 ± 2.3 | 4.0 ±  | 0.51 |
| ***72-hour experiments*** | | | |  |
| NADP, pmol/mg protein | Vehicle | 270.1 ± 105.6 | 329.4 ± 163.5 | 0.93 |
|  | 0.25 mM NR | 244.5 ± 94.9 | 255.0 ± 14.7 | 0.32 |
|  | 0.50 mM NR | 912.5  | 267.2 ±  | 0.99 |
|  | | | |  |
| NADPH, pmol/mg protein | Vehicle | 228.5 ± 227.2 | 101.5 ± 48.8 | 0.80 |
|  | 0.25 mM NR | 268.4 ± 198.8 | 144.7 ± 104.3 | 0.81 |
|  | 0.50 mM NR | 711.4 ±  | 171.8 ±  | 0.29 |
|  | | | |  |
| NADP:NADPH, ratio | Vehicle | 2.3 ± 1.7 | 3.2 ± 0.1 | 0.50 |
|  | 0.25 mM NR | 2.0 ± 2.0 | 2.4 ± 1.9 | 0.97 |
|  | 0.50 mM NR | 1.6 ± 1.1 | 1.6 ±  | 0.76 |
| ^1^ Repeated measures analyses using mixed effects models, unadjusted p-values. Analyses were performed between groups (i.e., *RYR1*-RM versus control myotubes).  *Adjusted p-value | | | | |

# **Supplementary Figures**

**Figure S1. Blood glutathione redox parameters evaluated by *RYR1*-RM mode of inheritance and participant age at time of baseline sample collection.** Results are presented for GSH (A and D), GSSG (B and E), and GSH:GSSG ratio (C and F). There was no significant difference in GSH, GSSG, or GSH:GSSG ratio between individuals with heterozygous versus compound heterozygous *RYR1*-RM as assessed by independent T-test following confirmation of normal data distribution (A-C). There was no significant correlation between participant age and GSH, GSSG, or GSH:GSSG in *RYR1*-RM affected individuals as assessed by Pearson correlation coefficient following confirmation of normal data distribution (D-F).

**Figure S2. Blood NAD^+^ redox parameters evaluated by *RYR1*-RM mode of inheritance and participant age at time of baseline sample collection.** In A-C data are presented as mean ± SD. Results are presented for NAD^+^ (A and D), NADH (B and E), and NAD^+^:NADH ratio (C and F). There was no significant difference in NAD^+^ or NADH concentrations between individuals with monoallelic versus compound heterozygous *RYR1*-RM (A-B). Compound heterozygous *RYR1*-RM affected individuals exhibited a significantly higher NAD^+^/NADH ratio versus monoallelic participants (C). Data were analyzed by independent T-test following confirmation of normal data distribution. There was no significant correlation between participant age and GSH, GSSG, or GSH/GSSG ratio in *RYR1*-RM affected individuals as assessed by Pearson correlation coefficient following confirmation of normal data distribution (D-F).

**Figure S3. Blood NADP redox parameters evaluated by *RYR1*-RM mode of inheritance and participant age at time of baseline sample collection.** In A-C data are presented as mean ± SD. Results are presented for NADP (A and D), NADPH (B and E), and NADP/NADPH ratio (C and F). There was no significant difference in NADP or NADPH concentrations, or NADP/NADPH ratio between individuals with monoallelic versus compound heterozygous *RYR1*-RM (A-C). Data were analyzed by independent T-test following confirmation of normal data distribution. There was no significant correlation between participant age and NADP, NADPH, or NADP/NADPH ratio in *RYR1*-RM affected individuals as assessed by Pearson correlation coefficient following confirmation of normal data distribution (D-F).

**Figure S4. Redox metabolite concentrations in *RYR1*-RM and control skeletal muscle tissue.** Data are presented as mean ±SD. Results for GSH redox analytes were below the limit of quantitation. (A-C) *RYR1*-RM affected individuals demonstrated significantly higher skeletal muscle NAD^+^ and NADH content versus controls but no difference in NAD^+^/NADH ratio. (D-F) *RYR1*-RM affected individuals demonstrated higher average skeletal muscle NADPH content versus controls, however this difference was not statistically significant, There was no difference in NADP or NADP/NADPH ratio between groups.

**Figure S5. Mitochondrial function in *RYR1*-RM and control myotubes pre- and post-nicotinamide riboside (NR) treatment.** Data are presented as mean ± SD. Cells were treated with a single low dose (0.25 mM) or high dose (0.50 mM) NR for 24 hours prior to analysis in triplicate using the Cell Mito Stress Test, per the manufacturer’s instructions (Agilent Technologies., CA, USA). Results were compared to control (untreated) myotubes derived from the same participants. **(A-B)** NR treatment appeared to result in a dose-dependent increase in maximal respiration and ATP production in *RYR1*-RM myotubes only (within group observation), and formal analyses were precluded by the limited sample size. Further experiments are warranted to confirm these observations, ideally utilizing a larger sample size. **(C-D)** There was no effect of NR treatment on spare respiratory capacity or mitochondrial coupling efficiency. **(E-F)** Basal oxygen consumption rate was notably higher in *RYR1*-RM cultures compared to otherwise healthy controls.

**Figure S6. Glutathione, NAD^+^, and NADP redox metabolite concentrations and ratios in *Ryr1* Y524S soleus muscle.** Data are presented as mean ± SD. There was no difference in redox metabolite concentrations between one-year old *Ryr1* Y524S mice and controls. GSH/GSSG, NAD^+^/NADH, and NADP/NADPH ratios were also comparable between strains.

**Figure S7. Glutathione, NAD^+^, and NADP redox metabolite concentrations and ratios in *Ryr1* Y524S whole blood.** Data are presented as mean ± SD. There was no difference in redox metabolite concentrations between one-year old *Ryr1* Y524S mice and controls. GSH:GSSG, NAD^+^:NADH, and NADP/NADPH ratios were also comparable between strains.
